# An American Text-Book of Physiology

**Published:** 1897-01

**Authors:** 


AN AMERICAN TEXT-BOOK OF PHYSIOLOGY. By Henry P. Bowditch, M. D., John G. Curtis, M. D., Henry H. Donaldson, Ph. D., W. H. Howell, Ph. D., M. D., Frederic S. Lee, Ph. D., Warren P. Lombard, M. D., Graham Lusk, Ph. D., W. T. Porter, M. D., Edward T. Reichert, M. D., and Henry Sewall, Ph. D., M. D. Edited by WILLIAM H. HOWELL, Ph. D., M. D., Professor of Physiology in the Johns Hopkins University, Baltimore, Md. Large octavo, pp. 1052. Illustrated. Price, cloth, $6.00; sheepskin and half-morocco, $7.00. For sale by subscription only. Philadelphia: W. B. Saunders, 925 Walnut street. 1896.

The general practitioner may be less familiar with the names of the foremost teachers of physiology of the day than with those of the leaders in other lines of medical work. The medical schools with which the contributors to this volume are associated show, however, their rank and authority. William H. Howell, Ph. D., the editor, is professor of physiology in Johns Hopkins University, and the Harvard Medical School, the Columbia University (College of Physicians and Surgeons), the University of Chicago, the University of Michigan, the Yale Medical School, the University of Pennsylvania and the University of Denver are represented in the contributors. The collaboration method has been tried successfully in other branches of medical science, and the writers give a demonstration of its advantages in handling the subject of physiology. As the preface ventures to hope, this method of treatment may prove to be the most attractive feature of the book.

The writers, for the most part, assume some knowledge of gross and microscopical anatomy on the part of the student, only introducing such descriptions as are needed to make clear the physiological question under discussion.

The chapters are signed, a feature which certainly adds to their interest. References, more or less full, to the literature of each topic are given, amounting in some cases to a complete bibliography. Those who wish to go deeper into the knowledge of a special subject will appreciate the plan.

Compared with the text-book of a decade or a hemi-decade ago, much that is new is seen; yet the physician who has read widely does not find here much with which he was not, at least in part, familiar. The volume sums up fairly the present state of our knowledge of physiology, and shows no less fairly the unknown territory of which our knowledge is but the borderland.

The section which the general practitioner will read with most profit is that on the central nervous system, while, as of old, the chapter on the special senses takes one into the fairyland of science. As a volume for the use of the average student of medicine, the book is too profound, a fault which reflects not on the work, and one which the steadily rising requirements for college entrance and training may overcome. The mechanical part of the book is excellent. M. J. F.

